# Sodium-hydrogen exchanger 6 (NHE6) deficiency leads to hearing loss, via reduced endosomal signalling through the BDNF/Trk pathway

**DOI:** 10.1038/s41598-020-60262-5

**Published:** 2020-02-27

**Authors:** Krystsina Kucharava, Yves Brand, Giuseppe Albano, Marijana Sekulic-Jablanovic, Andrea Glutz, Xunde Xian, Joachim Herz, Daniel Bodmer, Daniel G. Fuster, Vesna Petkovic

**Affiliations:** 1 Department of Biomedicine, and Clinic for Otolaryngology, Head and Neck Surgery, Hospital Basel, University of Basel, Basel, 4031 Switzerland; 2 Department of Nephrology and Hypertension, Inselspital, Bern University Hospital, and NCCR Transcure, University of Bern, Bern, Switzerland; 3 Center for Translational Neurodegeneration Research, University of Texas Southwestern Medical Center, Dallas, TX 75390 USA; 4 Department of Molecular Genetics, University of Texas Southwestern Medical Center, Dallas, TX 75390 USA; 5 Clinic for Otolaryngology, Head and Neck Surgery, Kantonsspital Graubünden, Chur, 7000 Switzerland

**Keywords:** Neuroscience, Auditory system

## Abstract

Acid-base homeostasis is critical for normal growth, development, and hearing function. The sodium–hydrogen exchanger 6 (NHE6), a protein mainly expressed in early and recycling endosomes, plays an important role in regulating organellar pH. Mutations in NHE6 cause complex, slowly progressive neurodegeneration. Little is known about NHE6 function in the mouse cochlea. Here, we found that all NHE isoforms were expressed in wild-type (WT) mouse cochlea. *Nhe6* knockout (KO) mice showed significant hearing loss compared to WT littermates. Immunohistochemistry in WT mouse cochlea showed that Nhe6 was localized in the organ of Corti (OC), spiral ganglion (SG), stria vascularis (SV), and afferent nerve fibres. The middle and the inner ears of WT and *Nhe6* KO mice were not different morphologically. Given the putative role of NHE6 in early endosomal function, we examined Rab GTPase expression in early and late endosomes. We found no change in Rab5, significantly lower Rab7, and higher Rab11 levels in the *Nhe6* KO OC, compared to WT littermates. Because Rabs mediate TrkB endosomal signalling, we evaluated TrkB phosphorylation in the OCs of both strains. *Nhe6* KO mice showed significant reductions in TrkB and Akt phosphorylation in the OC. In addition, we examined genes used as markers of SG type I (*Slc17a7*, *Calb1*, *Pou4f1*, *Cal2*) and type II neurons (*Prph*, *Plk5*, *Cacna1g*). We found that all marker gene expression levels were significantly elevated in the SG of *Nhe6* KO mice, compared to WT littermates. Anti-neurofilament factor staining showed axon loss in the cochlear nerves of *Nhe6* KO mice compared to WT mice. These findings indicated that BDNF/TrkB signalling was disrupted in the OC of *Nhe6* KO mice, probably due to TrkB reduction, caused by over acidification in the absence of NHE6. Thus, our findings demonstrated that NHEs play important roles in normal hearing in the mammalian cochlea.

## Introduction

The sodium–hydrogen exchangers NHE6 and NHE 9, members of the solute carrier (*SLC*) gene superfamily, are associated with endosomes. NHE6 is involved in early and recycling endosomes and NHE9 with late recycling endosomes^1,2^, with NHE6 also localizing to the cell surface. Variants of NHE6 (which is encoded at the Xq26.3 locus by *SLC9A6*) have been linked to features of Angelman syndrome in males, including ataxia, behavioural differences, epilepsy, intellectual disability, and microcephaly^3^. However, variants also have been identified that delineate three apparent subtypes outside of the classic Angelman presentation: two that are Angelman-like, including Christianson syndrome^4^, and a suite of features that includes autistic behaviours with severe intellectual disability, along with tau deposition and corticobasal degeneration^5,6^.

NHE6 is integral component of early, and recycling endosomes, and the cell surface in different phenotypes^7^. It is a candidate regulator of neuronal endolysosomal pathways. This protein has been suggested to regulate receptor recycling at the cell surface, along with endosomal volume and endosomal pH through sodium–hydrogen exchange^8–10^. Even after internalization, recycled receptors – including neurotrophin receptors – can continue to signal. The pH of endosomal populations represents a crucial regulator of how long these signals persist^11^, and the internal pH can be increased by proton exchange for cytoplasmic sodium or potassium, mediated by NHE proteins in the endosome.

Many members of the Rab family of small GTPases play central roles in compartment-specific processes; thus, they are commonly used as markers of vesicular identity^12,13^. The small GTPases, Rab5, Rab7, and Rab11, are key markers of early and late endosomes; these GTPases organize effector proteins into specific membrane subdomains^14–16^.

The regulation of pH creates a chain of associated events involving the kidney, bone, and inner ear that may triangulate on a cochlear role for NHEs. Although NHE activity in the inner ear remains something of a black box, NHEs 6 and 9 both are enriched in vestibular hair bundle plasma membranes, where they probably release H^+^ into the cytoplasm in exchange for K^+^. This process allows hair cells to dump protons in an ATP-independent manner following proton ingress via calcium pump activity^17^. At the kidney, coupling of bicarbonate reabsorption with acid secretion maintain the body's acid–base balance over the long term^18^, but in cases of failure, the result is distal renal tubular acidosis (dRTA). If acidosis persists, bone dissolution can follow, with attendant diseases such as osteomalacia or rickets. Progressive bilateral sensorineural hearing loss is a common comorbidity of dRTA^18^, possibly because of disrupted pH homeostasis in the inner ear^19^.

Tying these events together is the possibility that endosome acidification underlies some relevant neuronal defects. Neurotrophin signalling through brain-derived neurotrophic factor (BDNF) is required for central nervous system (CNS) development and maintenance^20^. Neuronal signalling using neurotrophin receptors (Trks) relies on endosomal pathways, which maintain neuron survival and correct targeting in the peripheral nervous system^21,22^. Survival of cochlear and vestibular neurons of the inner ear requires both BDNF and neurotrophin 3 (Nt3; now Ntf3), along with their receptors TrkB (Ntrk2) and TrkC (Ntrk3)^23^. In addition, these signalling molecules are responsible for outer hair cell (OHC) innervation^23^. NHE6 loss leads to smaller synapse populations and defects in dendrite and axon branching and dendritic spines, diminishing connectivity^24^.

In humans, sound perception has a major role in communication. Via mechanoreception, cochlear hair cells innervated by type I and II afferent neurons transduce incoming signals and pass them to the CNS through chemical synapses with the dendrites of spiral ganglion (SG) neurons^25,26^. Based on their molecular profiles, adult SG neurons are categorized into four types: three novel type I subclasses (Ia, Ib, and Ic neurons) and type II neurons^27^.

We hypothesized that the absence of NHE6 might disturb endosomal–lysosomal function, which might lead to a lack of BDNF/TrkB signalling within the cochlea and afferent cochlear nerve. Therefore, we examined the role of NHE6 in mouse cochlea by studying hearing loss in *Nhe6* knockout (KO) mice. We used various methods for assessing the consequences of hearing loss. We also studied secondary interruptions in BDNF/TrkB signalling and endosomal-lysosomal dysfunction. We next hypothesized that abnormal endosomal acidification might perturb endosomal signalling mechanisms relevant to neuronal development and arborization. We focused on endosomal-lysosomal dysfunction and found differences in Rab5, 7, and 11 expression in the SGs of WT and KO mice. In addition, we found that *Nhe6* KO mice had significant changes in gene expression specific to SG types I and II nerves and a reduction in cochlear afferent nerve fibres.

## Results

### *NHE1* – *9* genes are expressed in postnatal WT and *Nhe6 KO* mouse cochleae

**Figure 1:**
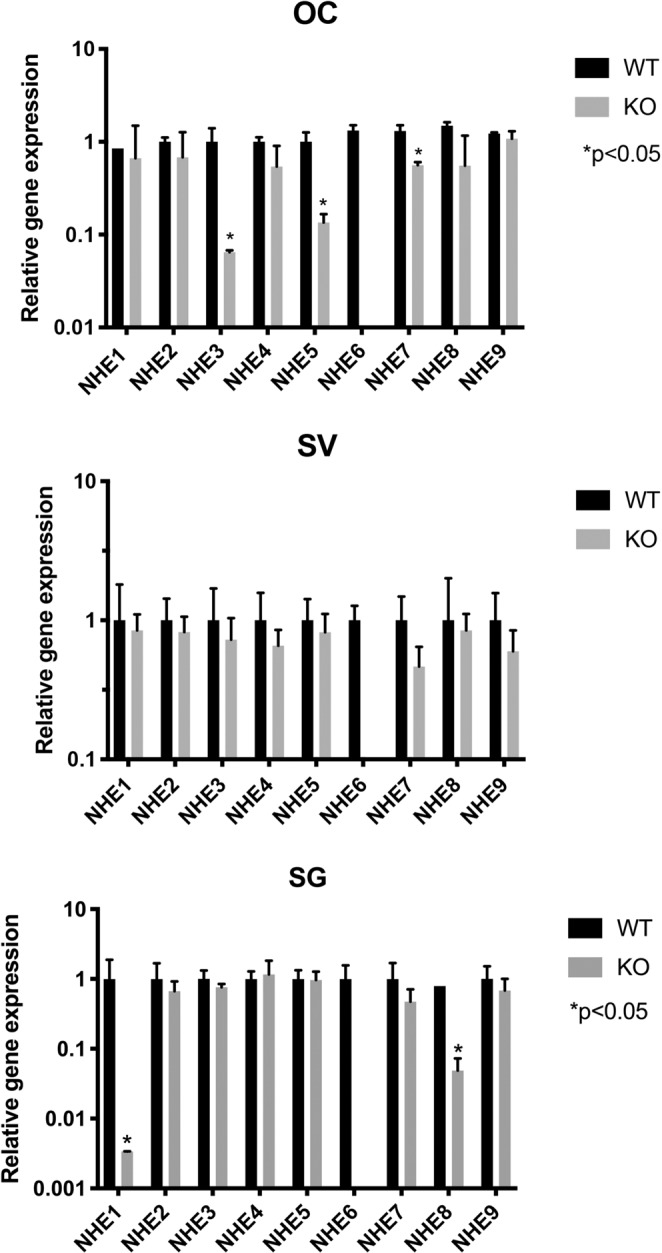
Quantitative PCR results show SLC9A subgroup genes, *Nhe1* – *9*, expressed in the cochlea of 5-day-old WT and *Nhe6* KO mice. (*Top*) In OC samples, *Nhe3, 5*, and *7* expression levels were significantly down-regulated in the OCs of KO compared to WT mice^56^. In SV samples, no significant difference is observed between WT and KO mice. (*Bottom*) In SG samples, only in *Nhe1* and *Nhe8* was down-regulated in KO compared to WT mice. Results are the mean fold-change in transcript levels ± SD, compared to GAPDH, a housekeeping gene. qPCR was done in triplicate (n = 20 mice and 40 OC per strain was pooled) *p < 0.05 (Student’s t-test).

The NHE proteins belong to a large family of transporters known as the solute carrier (SLC) gene superfamily^28,29^. We performed quantitative PCR (qPCR) to investigate *NHE* expression in cochlear samples from postnatal day 5 (P5) WT and *Nhe6* KO mice. We found that all *NHE* isoforms were expressed in the inner ears of both strains (except *NHE6* KO mice lacked *Nhe6*). However, *Nhe3*, *Nhe5*, and *Nhe7* expression in the OC, and *Nhe1* and *Nhe8* expression in the SG were down-regulated in *Nhe6* KO mice, compared to WT mice (Fig. 1). Considering these differences in gene expression, we have tested the protein expression levels of NHE3 and NHE6 and found no significant differences between WT and NHE6 KO (Supplementary Fig. 2).

### Adult *Nhe6 KO* and WT mice show similar cochlear microanatomy

**Figure 2:**
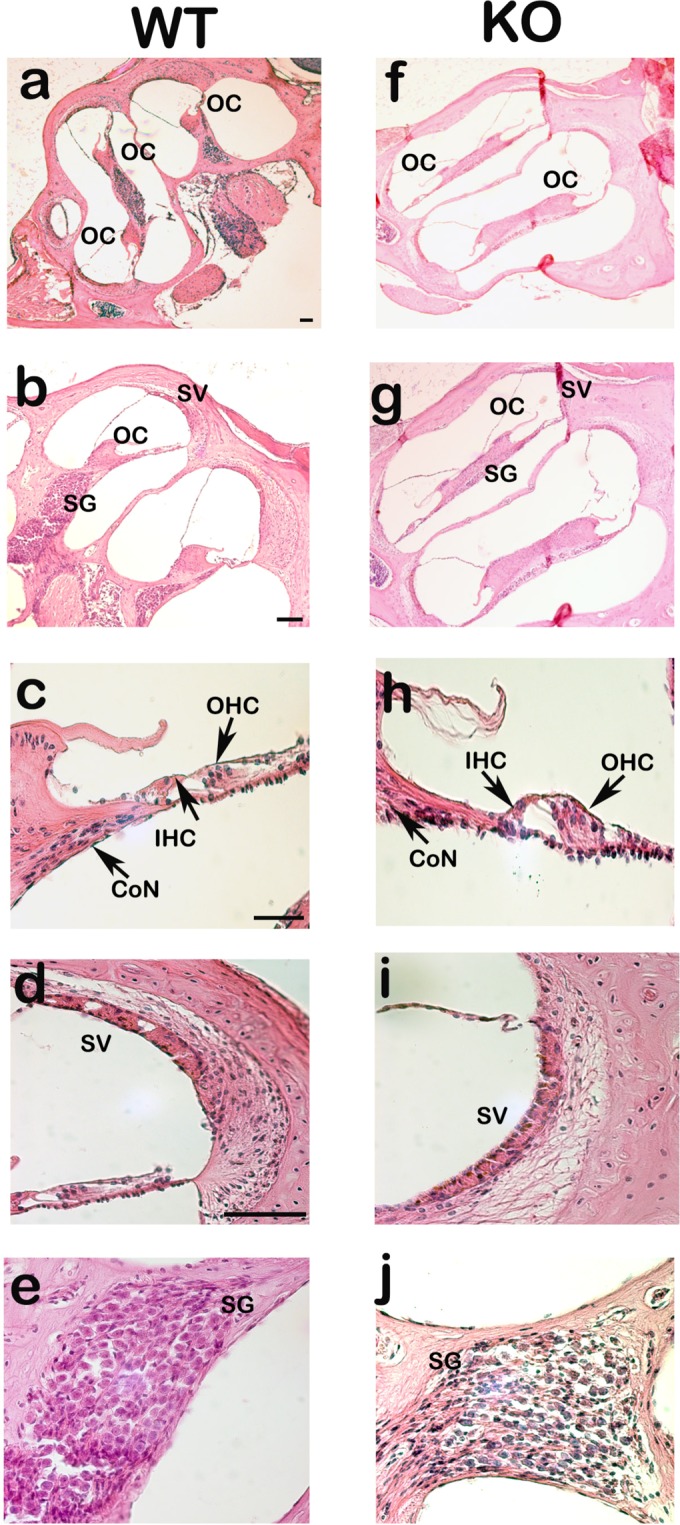
Light microscopy images of WT (**a**–**e**) and *Nhe6* KO mice (**f**–**j**) mouse cochleae (sagittal sections) stained with haematoxylin/eosin. No differences were observed in the morphology of WT and *Nhe6* KO mice. Arrows and abbreviations indicate the locations of inner hair cells (IHC), outer HCs (OHC), the spiral ganglion (SG), cochlear nerve (CoN) and stria vascularis (SV). (n = 3 mice per strain). Scale bars = 50 µm.

Recently, physiological and pharmacological studies have indicated that NHEs participate in ion homeostasis in the inner ears of guinea pigs and gerbils^30,31^. We stained sections of the temporal bones of adult WT and *Nhe6 KO* mice with haematoxylin and eosin to study potential differences in cochlear microanatomy. We found no significant differences in inner ear microanatomy between WT (Fig. 2a–e) and *Nhe6 KO* mice (Fig. 2f–j). All displayed OCs with three rows of OHCs and one row of inner hair cells (IHCs). The basilar membrane, SG neurons, and the SV also showed similar morphology between mouse strains. Finally, the middle ear microanatomy was not significantly different between WT and *Nhe6 KO* mice (Supplementary Fig. 1).

### NHE6 protein expression patterns in adult mouse cochlea

**Figure 3:**
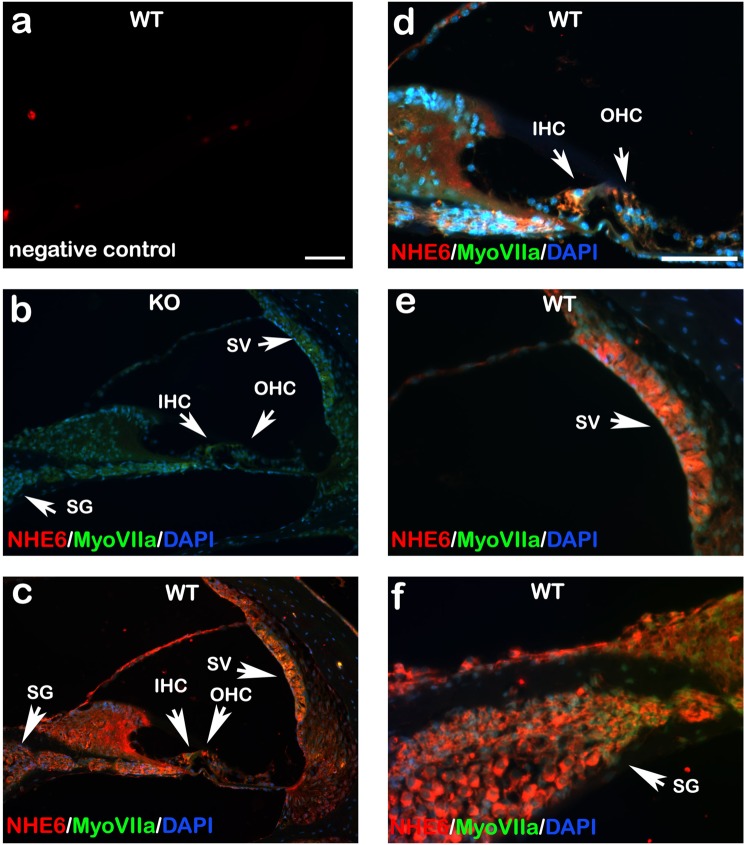
Immunohistochemistry images show NHE6 protein expression patterns in adult mouse cochleae. Cell nuclei are stained blue and NHE6 is stained red. Myosin VIIa (green) served as a marker for hair cells. (**a**) Cochlear section stained only with secondary antibodies (negative control). (**b**) Cochlea from a *Nhe6* KO mouse shows no NHE6 expression. (**c**) NHE6 is abundant in the cochlea of a WT mouse. NHE6 is located in the SV and SG. (**c**,**d**) Strong labelling is observed in or around the IHC and OHC of the WT mice. (**e**) NHE6 expression in the SV at higher magnification. (**f**) NHE6 expression in the SG at higher magnification. OC, organ of Corti; SV, stria vascularis; SG spiral ganglion; IHC, inner hair cells; OHC, outer hair cells; (n = 3 mice per strain). Scale bars 50 µm.

We first investigated the expression and distribution of NHE6 in the mouse cochlea. Previously, NHE activity was demonstrated in the guinea pig cochlea^32^. We stained formalin-fixed paraffin-embedded adult mouse cochlear sections with specific fluorescently-labelled antibodies against NHE6. Cochlear section of WT mice stained only with secondary antibodies served as negative control (Fig. 3a). As expected, we detected no NHE6 staining in KO mouse cochleae (Fig. 3b). In WT mouse cochleae, the NHE6 protein was found in the IHCs, OHCs, SV, and SG (Fig. 3c). Higher-magnification images revealed strong NHE6 staining in IHCs and OHCs (Fig. 3d). NHE6 staining was also detected in the SV and SG (Fig. 3e,f). Split single channel exposure to myosin VIIa, DAPI and NHE6 could be found in Supplementary Fig. 3.

### Adult NHE6 KO mice show elevated auditory thresholds compared to WT littermates

**Figure 4:**
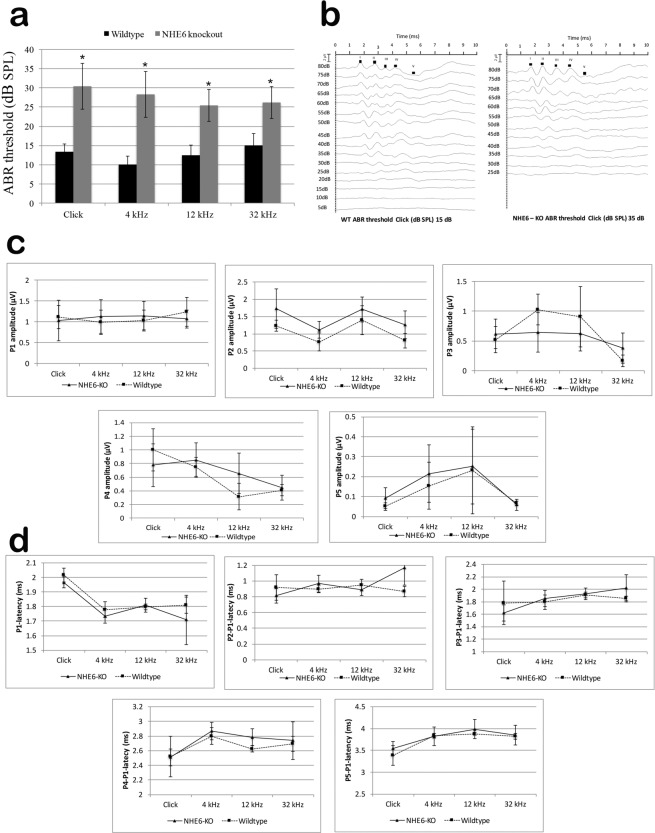
Click-induced ABR thresholds in WT and *Nhe6* KO mice at 3 months of age. (**a**) Clicks and frequency-specific (4 kHz, 12 kHz, and 32 kHz) sounds were delivered to determine ABR thresholds in 5 WT and 7 *Nhe6* KO animals. Bars represent mean ± standard deviation. Click-induced ABR thresholds were significantly lower (about 15 dB; SPL) in WT mice than in *Nhe6* KO mice (about 35 dB; SPL) *p < 0.05 (ANOVA). (**b**) Representative examples of click-induced ABRs in WT and *Nhe6* KO. The peaks are indicated in Roman numerals (I–V). (**c**,**d**) Sounds 40 dB above the threshold at each frequency were delivered to WT and *Nhe6* KO mice. Plots show (**c**) peak amplitudes and (**d**) latencies at different points on the ABR waveforms (P1-P5). The P1-P5 amplitudes were not significantly different between WT and *Nhe6* KO mice. The raw latency shown for P1 and the corrected latencies of P2-P5 (corrected for P1 latency) were not significantly different between WT and *Nhe6* KO mice. Error bars represent the mean ± standard deviation.

To extend these studies, we tested hearing function *in vivo* in WT and *Nhe6* KO mice. Mice were tested with broadband click stimuli and pure tones to determine hearing thresholds. *Nhe6* KO mice exhibited significantly elevated auditory brainstem response^33–36^ thresholds for click stimuli and sound stimuli delivered at 4 kHz, 12 kHz, and 32 kHz frequencies, compared to WT littermates (Fig. 4a). Representative click-induced ABR recordings from WT and *Nhe6* KO mice are shown in Fig. 4b. No differences were observed in the ABR waveforms, amplitudes (Fig. 4c), or inter-peak latencies (Fig. 4d) between WT and *Nhe6* KO mice.

### Phospho-Trk and Akt levels are reduced in *Nhe6* KO, compared to WT mice

BDNF regulates many facets of central neurons, including neuronal survival and differentiation, neuronal growth, synaptogenesis, plasticity, and the maintenance of neuronal circuits^37^. BDNF mainly mediates neuronal growth effects through its tyrosine kinase receptor, TrkB^38^. Activation first causes TrkB dimerization and autophosphorylation, which then leads to the activation of the three main signal pathways: Ras/Raf/MAPK, PI3K/AKT, and PLC-γ^39^. Neurotrophin receptors use endosomal pathways for signalling in neurons. Post-endocytic trafficking of endocytic receptors are mainly regulated by Rab monomeric GTPases. Rabs regulate vesicular trafficking by controlling the transport, anchoring, and coupling of vesicles through effector binding^40^. Rab5, Rab7, and Rab11 are among the key GTPases known to be involved in BDNF/TrkB signalling^41^.

**Figure 5:**
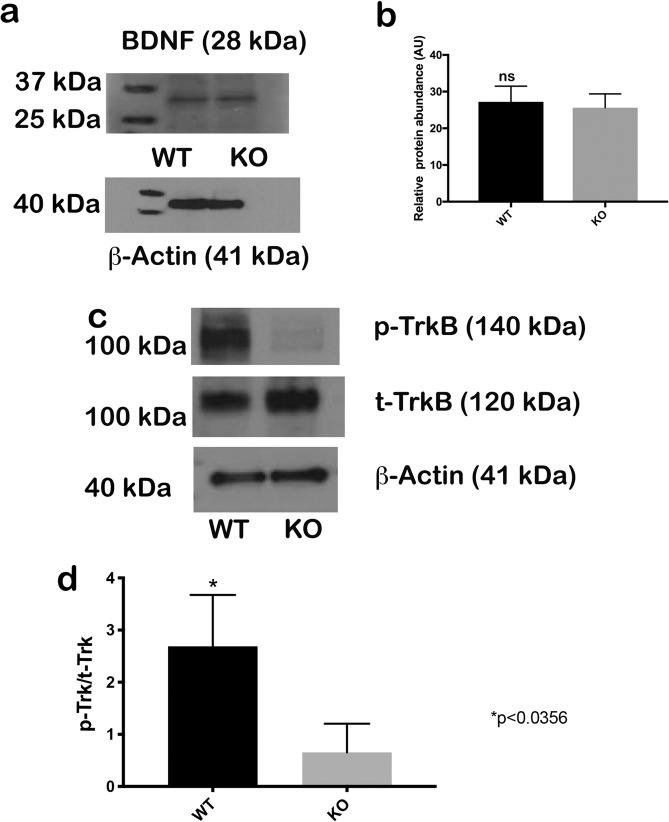
Western blot detection of BDNF, phosphorylated TrkB (p-Trk), and total TrkB (t-Trk) proteins in the OCs of WT and *Nhe6* KO mouse cochleae. BDNF, p-Trk, and t-Trk proteins were detected in extracts from the OCs of WT and KO P5 mice. (**a**) BDNF levels were the same in both samples. (**b**) Quantification of Western blot signals show BDNF protein expression. Values are the mean ± SD expression levels relative to endogenous β-actin. (**c**) p-Trk levels were reduced in KO samples, compared to WT samples. t-Trk levels were similar in the two mouse strains. (**d**) Quantification of Western blot signals show the ratio of p-Trk/t-Trk signals. Values are the mean ± SD, normalized to ß-actin. n = 10 mice per strain, 20 explants per mouse group; *p < 0.035.

We detected no significant difference in BDNF protein contents between KO and WT mice (Fig. 5a). Quantification of Western blot signals show BDNF protein expression in OC explants of KO and WT mice (Fig. 5b). Western blot analyses indicated that phosphorylated TrkB (p-Trk) levels in the OC of *Nhe6* KO mice neurons were significantly reduced compared to WT mice, while levels of total TrkB (t-TrkB) remain similar (Fig. 5c). These findings suggested that the absence of NHE6 promoted significant differences between WT and KO mice (Fig. 5d).

**Figure 6:**
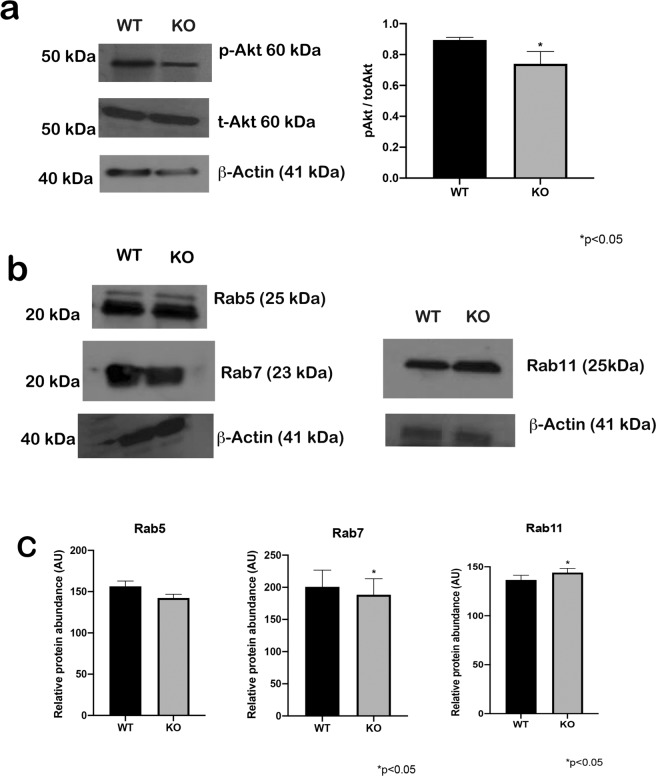
Western blots show expression of Akt and Rab5, 7, and 11 in the OC of WT and *Nhe6* KO mice. (**a**, *Left*) Phosphorylated Akt (p-Akt) was reduced in KO samples compared to WT samples. (*Right*) Quantification of Western blot signals show the mean expression of p-Akt levels normalized to total Akt (t-Akt) levels. (**b**) Rab5 expression is the same in the both samples. In contrast, Rab7 is reduced and Rab 11 is elevated in *Nhe6* KO compared to WT mice. (**c**) Quantification of Western blot signals show mean expression levels normalized to β-actin levels. n = 20 mice per strain and including pups of both sexes., 40 OCs per mouse group, p < 0.05 with the Student’s t-test.

To investigate Akt activation, we performed Western blots with OC lysates from WT and KO animals. The phosphorylated Akt signal (p-Akt) was significantly lower in the OC of KO mice compared to WT mice (Fig. 6a), indicated by the ratio of p-Akt to total Akt (t-Akt). To characterize the NHE6 endosomal compartment further, we quantified the expression of Rab5 (early endosomes), Rab7 (late endosome), and Rab11 (recycling endosome). Rab5 expression was not significantly different between the strains. Furthermore, Rab7 protein levels were reduced and Rab11 levels were increased in *Nhe6* KO mice compared to WT littermates (Fig. 6b,c).

### Gene expression in SG neurons I and II in WT and *Nhe6* KO mice

**Figure 7:**
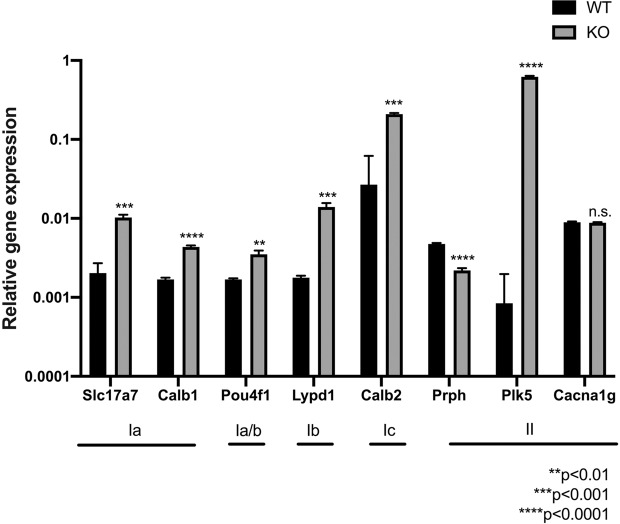
Quantitative PCR results show expression of specific markers for SG neurons, types Ia-Ic and II, in WT and *Nhe6* KO mice. To elucidate the mechanisms underlying the NHE6 influence on the BDNF-Trk signalling pathway, which regulates neuronal growth and branching, we exam  ined gene expression in SG neurons. Type I neurons were identified with specific genetic markers for 3 subclasses: *Slc17a7*, *Calb1*, *Pou4f1*, and *Cal2*. Type II neurons were identified with 3 specific genetic markers: *Prph*, *Plk5*, and *Cacna1g*. Almost all genes are significantly up-regulated in *Nhe6* KO mice SGs compared to WT SGs. Values are the mean ± SD, n = 20 mice per strain and 40 OC per mouse group including pups of both sexes. **p < 0.01; ***p < 0.001; ****p < 0.001; n.s.: not significantly different.

Petipré *et al*. identified four distinct types of adult SG neurons, including three novel subclasses of type I neurons (Ia, Ib, and Ic neurons) and type II neurons. These types can be distinguished based on unique and combinatorial molecular profiles^27^. We next explored the expression of specific genes in types I and II SG neurons dissected from WT and *Nhe6* KO cochleae. We performed qPCR in three biological replicates to investigate differences in gene expression. SG II neurons were specifically identified by the expression of peripherin (*Prph*), *Plk5*, and *Cacna1g*. SG Ia neurons were identified by the expression of *Slc17a7, Calb1*, and *Pou4f1*. SG Ib neurons were identified by the expression of *Lypd1* and *Pou4f1*. SG Ic neurons were identified by the expression of *Calb2*. All these genes, except *Cacna1g*, were differentially expressed in *Nhe6* KO mice, compared to WT mice (Fig. 7).

### *Nhe6* KO mice exhibit a reduction in afferent cochlear neurons

**Figure 8:**
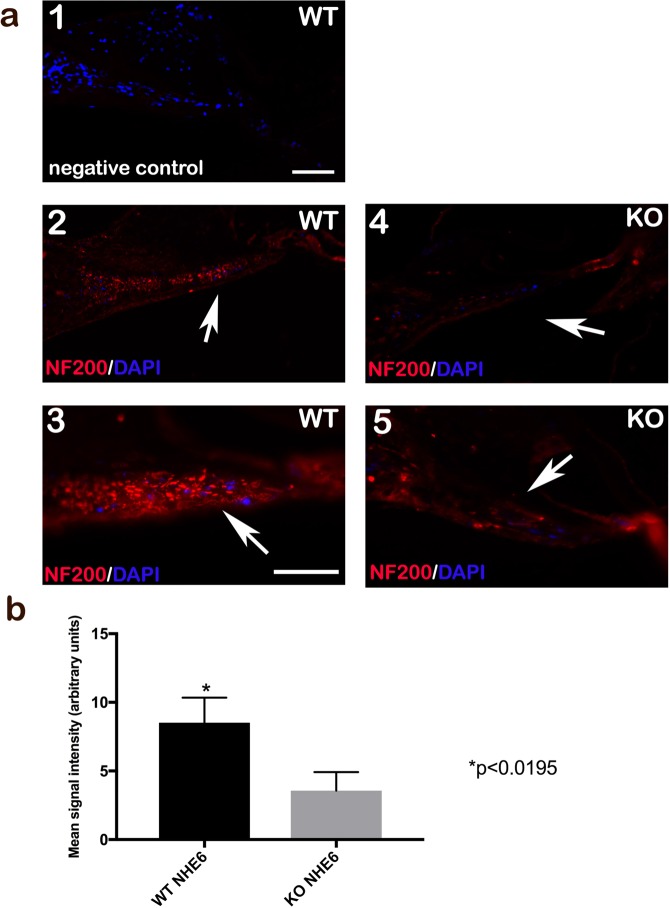
Immunofluorescence microscopy detection of NF200 in the cochleae of WT and *Nhe6* KO mice. (**a**) Cochlear sagittal sections of adult WT (*left*) and *Nhe6* KO (*right*) mice labelled with anti-NF200 antibody (a-1) Negative control (in WT), blue staining is DAPI. (a-2,3) Visualization of afferent innervations with red NF200 staining in WT. (a-4,5) Visualization of afferent innervations with red NF200 staining in KO. Blue: nuclear marker (DAPI). Arrows point to neuronal fibres in cochlear nerves. (n = 3 mice per strain) Scale bars = 50 μm. (**b**) Quantifications of fluorescence intensities indicate the abundance of fibres with detectable neurofilaments in the cochlear nerves. WT mice had significantly more fibres than *Nhe6* KO mice. Values are the mean ± SD *p < 0.0195.

Neurotrophin signalling controls cochlear innervation. A lack of neurotrophin signalling in the cochlea has been well documented in early postnatal animals. This condition results in the loss of cochlear sensory neurons and a region-specific reduction in target innervation along the tonotopic axis^42^. Disruptions in BDNF/Trkb signalling lead to impaired neural growth. Here, we sought to evaluate neural growth by staining cochlear sagittal sections of WT and *Nhe6* KO adult mice with an anti-neurofilament (anti-NF200) antibody to visualize afferent innervations (Fig. 8a). Signal intensity quantifications revealed a significant reduction in NF200-positive fibres in the cochlear nerves of *Nhe6* KO mice, compared to observations in WT mice (Fig. 8b).

## Discussion

NHE6 participates in regulating cytosolic and organellar pH and cell volume. NHE6 also contributes to whole body volume and acid–base homeostasis. Mutations in the *NHE6* genes cause neurological disease and contribute to the pathophysiology of multiple human diseases^3^. Little is currently known about NHEs in the inner ear. In this study, we investigated the expression and function of NHE6 in mouse cochlea with the *Nhe6* KO mouse. We found that NHE6 mRNA was expressed in all compartments of the mouse cochlea (OC, SG, SV). These results were consistent with those from studies performed in guinea pigs and gerbils^30,31^. Despite the fact that we did not find any morphological differences in the middle or inner ears of WT and *Nhe6* KO mice, the *Nhe6* KO mice displayed significant hearing loss compared to WT littermates. *Nhe6* KO mice had elevated hearing thresholds in click-induced and frequency-specific ABR measurements, compared to WT littermates. The ABR measurements were, on average, about 15 dB different between *Nhe6* KO and WT mice, in the acoustic spectrum of 4 kHz to 32 kHz. Interestingly, our data did not show any differences in the latencies or amplitudes of the evoked waves between strains. This finding suggested that the auditory stimuli could travel normally along the successive nuclei in the central auditory pathway, once sound levels surpassed the elevated threshold. These findings indicated that hearing loss in *Nhe6* KO animals was due to cochlear damage.

As noted, NHE6 loss can lead to a too-steep decrease in endosomal pH and reduced signalling^24,43^ which in turn can reshape neuronal arborization through changes in endosomal neurotrophin signalling^44–46^. Embryonic cochlear innervation relies on neutrophins and their respective Trks^42,47^, and in model organisms, cochlear sensory neurons are lost without appropriate signalling. Loss of signalling also is associated with region-specific reductions in innervation, decreases that track with the axis of frequency detection^42^. Here, mice lacking Nhe6 had reduced levels of phosphorylated Trk and Akt proteins in the OC compared to WT animals, yet knockout animals had no differences in BDNF protein levels versus WT mice. BDNF, like other neutrophins, is secreted from both the soma and distal neuronal regions, so that signalling to the nucleus can be either local or long distance. One group proposed that the OC does not itself produce BDNF but rather acquires it via the systemic circulation, explaining how levels could still be the same despite strain differences^47^. The PI3K/AKT signalling pathway controls neuronal growth through its effects on axonal and dendritic protein production and cytoskeleton dynamics^38^, and NHE6-associated endosomes have been identified in growing axons and dendrites^24^.

To characterize the NHE6 endosomal compartment in mouse cochlea further, we quantified the levels of Rab5 (early endosomes), Rab7 (late endosomes), and Rab11 (recycling endosomes) in the OCs of both strains. Rab5, 7, and 11 were previously shown to be important for normal neuronal migration and maturation in the cortex, through the regulation of N-cadherin trafficking^48^. That finding revealed that the endocytic pathway played a physiological role in development. We found that Rab5 expression was similar in both strains, but the level of Rab7 protein was reduced in KO, compared to WT animals. These two endosomal proteins mediate endocytosis and promote neuronal growth through BDNF/Trk signalling. The reduction in Rab7 signalling was a consequence of interrupted signalling. In contrast, Rab11 expression was increased in KO samples, compared to WT samples. This result might be explained by findings from Rink *et al*., who showed that Rab11 was overexpressed in injured neurons, which led to neuronal autophagy. That study showed that neuronal exosomes enriched with miR-21-5p could inhibit neuronal autophagy by targeting Rab11a, which suppressed trauma-induced, autophagy-mediated nerve injury *in vitro*^13^.

Auditory nerve sound signal processing was hypothesized to originate from the diverse biophysical properties of class I and II SG neuronal fibres. Therefore, we examined the expression of specific markers for types I and II SG neurons. Petipré *at al* identified four types of SG neurons (i.e., three type I subclass neurons and one type II class), along with numerous new marker genes. They also described a comprehensive genetic framework that could shape synaptic communication patterns. In addition, they characterized the differential projection patterns of distinct of type I subclasses to hair cells^27^. In our study, genes specific to both types I and II neurons were up-regulated in the SGs of *Nhe6* KO mice. In contrast, immunohistochemistry showed a reduced number of neuronal fibres in the cochlear nerve of *Nhe6* KO mice. Additionally, mouse brains with disrupted *NHE6* gene displayed reduced axonal and dendritic branching, reduced synapse numbers, and reduced circuit strength^24,49^. We assumed that these findings might be explained by the fact that the SG samples were derived from postnatal mice, and there is evidence that perinatal neuronal diversification occurs before the onset of hearing^27,50^. We showed that BDNF/Trk signalling was impaired in the cochlea of *Nhe6* KO mice, which resulted in reductions in neuronal production and development. We found that the other genes described by Petipré *et al*. were up-regulated in the present study, which might reflect a compensatory effect in the SG of *Nhe6* KO mice.

In conclusion, our study showed that *Nhe6* KO mice displayed significant, moderate hearing loss. NHE6 was expressed in all WT cochlear tissues. Our findings indicated that the depletion of NHE6 had an effect on Trk protein turnover and endosomal signalling. These results suggested that the failure of dendritic and axonal growth and branching in *NHE6* KO mice might have occurred, in part, due to deficiencies in BDNF/TrkB signalling. This novel finding suggested that NHE6 is important for normal hearing and function.

## Materials and Methods

### Animal care and handling

We isolated cochleae from *Nhe6* KO mice and their WT littermates. Steven Walkley of Albert Einstein College of Medicine (Bronx, NY) provided animals with a targeted *Nhe6* gene disruption (Jackson Laboratories Stock# 005843, strain name B6.129P2-Slc9a65tm1Dgen). All mice described here were backcrossed for >10 generations into a *C57bl6/j* (WT) background. We performed genotyping as described in Strømme *et al*.^51^. Animals had *ad libitum* access to water and standard mouse diet and were maintained under 12-h light/12-h dark at the animal experimental station in the Department of Biomedicine Research, which provides housing, feeding, and in-house breeding for colony maintenance. After a week of acclimation, we placed one male with two females in a single cage for breeding. For this work, we used both adult and postnatal-day-5 mice.

All animal procedures were conducted in compliance with the European Communities Council Directive of 24 November 1986 (86/609/EEC) and approved by the Kantonales Veterinäramt, Basel, Switzerland. Animals were inspected regularly for health status, and maintenance protocols were subject to the provisos of the animal welfare ordinance.

### Cochlear microdissection

For the microdissections, we decapitated P5 *Nhe6* KO and WT male and female mice and performed microdissection under light microscopy to isolate the OC, SV, and SG. Tissues were placed initially in ice-cold PBS, and OCs were prepared as described^52^.

### RNA isolation and quantitative PCR

Tissues for RNA isolation were placed in RNAlater (Ambion, USA), and RNA was isolated as described^53^, using the Direct-Zol RNA MiniPrep kit (Zymo Research, USA) and following manufacturer instructions. RNA quantity and quality were determined using a NanoDrop 1000 (Thermo Scientific), and all samples had 260/280-nm absorbance ratios between 1.8 and 2.1. Reverse-transcription of total RNA (1000 ng) was performed using the High-Capacity cDNA Reverse Transcription Kit (Applied Biosystems, USA), and qPCR was run with the ABI Prism 7900HT Sequence Detection System (Applied Biosystems, USA) and with Power Sybr Green Master Mix (Applied Biosystems, USA) or TaqMan protocol (for NHE gene expression). qPCR primers (250 nM per reaction) were obtained from Microsynth (St. Gallen, Switzerland) and are listed in Table 1 in the supplementary information. The cycling steps were as follows: 10 min at 95 °C, followed by 40 cycles of 95 °C for 15 s and 60 °C for 60 s. To control for nonspecific amplification and contamination, we ran template-free controls. We used the comparative threshold cycle method to calculate relative cDNA quantities, with GAPDH as reference.

### Western blotting

We have previously described preparation of protein samples from the cochlea of P5 pups^53^. Briefly, samples were placed in cell lysis buffer with a protease inhibitor cocktail (Sigma C3228, P8340), followed by homogenization on ice for 1 min. We used the NanoDrop 1000 (Thermo Scientific) to measure proteins, with mouse brain lysate serving as control. Lysates then were mixed with Laemmli sample buffer (Sigma) in equal amounts and heated for 5 min at 95 °C. After sample resolution on SDS-PAGE gels (10 μg protein per lane), gels were blotted onto polyvinylidene fluoride membranes. Following blockage of nonspecific sites with 5% nonfat dry milk in PBS for 1 h at room temperature (RT), we incubated membranes with primary antibodies in PBS-Tween. Primary antibodies were as follows: mouse monoclonal anti-p-TrkB (1:1000, Santa Cruz Biotechnology, sc-8058), mouse monoclonal anti-t-TrkB (1:1000, Invitrogen, MA5-14903), rabbit polyclonal anti-p-Akt 1:1000 (Cell Signalling, #9275), rabbit polyclonal anti-t-Akt 1:1000 (Cell Signalling, #9272), rabbit polyclonal anti-BDNF (1:1000, Sigma AG, AB1779SP), and rabbit monoclonal anti-Rab5, 7, 11 (1:1000, Cell Signalling, #3547; #9367; #5589). Secondary antibodies were anti-mouse (1:3000, Cell Signalling, #7076P2) and horseradish peroxidase–linked anti-rabbit (1:2000, Cell Signalling, #7074P2). Membranes were incubated overnight with primary antibodies at 4 °C, washed with PBS-Tween (3 × 10 min), and incubated with an appropriate secondary antibody at RT for 1 h. After washings, Super Signal West Dura Extended Duration Substrate (Thermo Scientific, Switzerland) was used to visualize bands, with rabbit polyclonal β-actin (1:1000, Cell Signalling, #4967) to control for protein loading.

### Paraffin cochlear sections for morphological staining and immunofluorescence

To assess middle and inner ear morphology and HNE6 and N200 expression levels, we used cochleae from adult male and female WT and Nhe6 KO mice. After animals (n = 4) were killed via sodium pentobarbital (100 mg/kg), they were transcardially perfused (50 ml phosphate-buffered 4% paraformaldehyde, pH 7.4; 4 °C). The inner ear was removed and decalcified for 10 days in a light-protected flask containing 120 mM EDTA (Merck, New Jersey, USA) in distilled water (pH 6.8). Cochleae then were dehydrated in graded ethanols (70, 80, 95, and 3 × 100%, 1 h each; 3× xylol for 1 h; 2× Paraplast at −60 °C for 1 h; and 1× Paraplast at −60 °C for 10 h), following by paraffin embedding at 56°C.

For histological evaluations, 10-µm sections were made using a Leitz microtome and mounted on Superfrost-Plus slides (Menzel, Braunschweig, Germany). Immunohistochemistry has been described before^54^, but briefly, sections were deparaffinized, rehydrated, and washed in PBS for 5 min, followed by incubation in blocking solution (TBS: 50 mM Tris, 0.9% NaCl; 0.5% Triton X-100, pH 7.35; 3% normal goat serum [NGS]) for 1 h at RT. For antibody binding, we incubated the sections with a mouse monoclonal antibody against myosin VIIa (1:350, Abcam, ab150386), primary anti-NF200 (1:500, Sigma AG, N4142), and rabbit anti-NHE6 (1:1000). To generate a rabbit polyclonal antibody targeting the NHE6 carboxyl terminus, we used cysteine bonding to couple maleimide-activated KLH with a synthetic peptide containing the last 13 amino acids of mouse NHE6. Rabbit immunization took place at UT Southwestern, following IACUC-approved protocols^55^. After overnight dilution of antibodies in TBS with 1% NGS at 4 °C and then three TBS washes, sections were incubated with the appropriate Alexa-conjugated secondary antibodies (1:250; Molecular Probes, Lubio Science, Switzerland). Antibodies were diluted in TBS with 1% NGS, and incubations were done at RT for 2 h. Following another TBS wash and DAPI counterstain, sections were mounted on glass slides using Mowiol. Negative controls were samples of WT tissues. To visualize binding, we used an Olympus AX-70 microscope equipped with a spot digital camera and adjusted recorded images for brightness and contrast using Image-Pro Plus and Photoshop.

Haematoxylin and eosin staining also was performed on some paraffin-embedded sections. As we previously described^52^, haematoxylin staining (Sigma Aldrich, Switzerland) was performed on sections for 5 min, followed by a 15-min rinse in tap water and immersion in eosin 1% aqueous (Eosin Y disodium salt, in deionized water; Sigma Aldrich, Switzerland) for 1–2 min. Before use, the solutions were filtered (Baxter grade 363, qualitative), and sections were rinsed in tap water until the water was clear. After dehydration in ascending alcohol concentrations (50%, 70%, 80%, 2× 95%, and 2× 100%), xylene clearance (2 times), and mounting with Eukitt (Fluka, Sigma Aldrich, Switzerland), sections were imaged using an Olympus IX50 microscope with a spot digital camera. As above, images were adjusted for brightness and contrast using Image-Pro plus and Photoshop.

### Hearing function tests

We administered the ABR test to all animals before treatment, as described previously^53^ (Tucker-Davis Technologies; TDT -RZ6-A-P1 hardware and software; Alachua, FL, USA), followed by their anesthetization with intraperitoneal ketamine (80 mg/kg body weight [BW]; Graeub AG, Switzerland), 12 mg/kg BW xylazine (Graeub AG, Switzerland), and 2 mg/kg BW acepromazine (Arovet AG, Switzerland). Testing was performed in sound-attenuating acoustic chambers, with infrared warming pads (Kent Scientific Corporation, USA) to keep animal body temperature at 38 °C. BioSig software was used to synthesize the tone burst acoustic stimuli (duration, 10 ms; rise/fall time, 0.5 ms; Blackman window), with an SA1 audio amplifier and a Multi Field Speaker-Stereo (MF1-S) as transducer. For testing, we placed an inverting electrode over the mastoid of the target ear, a non-inverting needle electrode at the midline vertex, and a ground electrode on the upper hindlimb. The speaker (MF1-S) was placed in the ear canal. Electrodes collected the signals, which then were amplified 20× with band-pass filters (100 Hz to 5 kHz) and input into a real-time processor (RA4PA) for software processing (BioSig RP software; TDT). We evaluated thresholds at 4, 12, or 32 kHz. Signals were preamplified with a gain of 20, and for each trial, we averaged 1000 sweeps. The starting level for stimuli presentation was 90 dB, which was incrementally decreased by 5 dB to threshold (the lowest intensity at which a visible, repeatable ABR wave could be observed in two averaged runs) and the ABR wave disappeared for each frequency. We calculated the amplitude and latency growth input–output function slopes (i.e., amplitude and latency as a function of stimulus intensity), as described before^53–55^.

### Statistical analysis

Statistical analyses were performed with the Student's t-test for unpaired samples. When more than two groups were compared, we performed a one-way analysis of variance (ANOVA), followed by the Bonferroni post-hoc test. A p value <0.05 was considered statistically significant. All statistical tests were two-sided. Data were analysed with GraphPad Prism 7 software (La Jolla, CA, USA).

## Supplementary information

Supplementary information.
