# Supplementary figures and images for: Sodium-hydrogen exchanger 6 (NHE6) deficiency leads to hearing loss, via reduced endosomal signalling through the BDNF/Trk pathway
Source: Sci Rep. 2020 Feb 27;10:3609. doi: 10.1038/s41598-020-60262-5 (PMC7046661; doi:10.1038/s41598-020-60262-5)

Supplementary: WT negative control to Figure 3

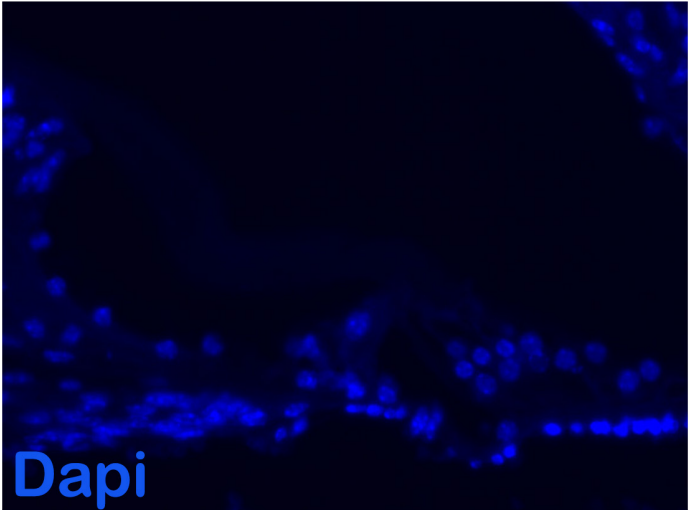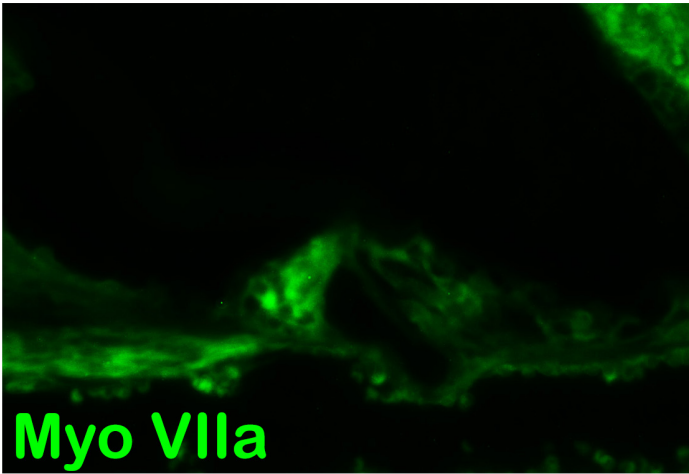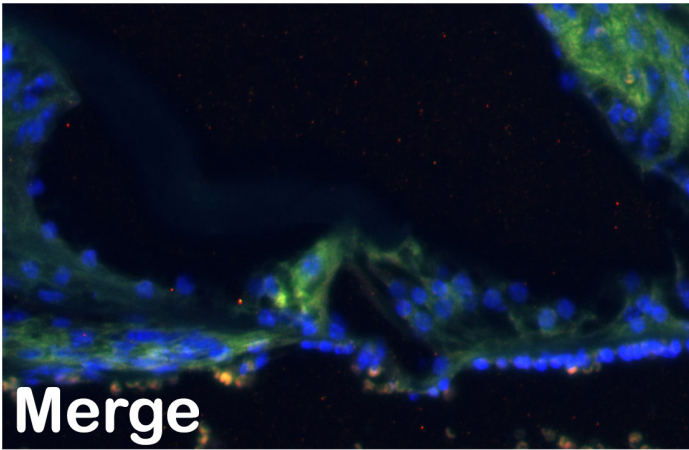

Supplement: Supplementary file 1 — Supplementary information. [file 41598_2020_60262_MOESM1_ESM.pdf]
